# Analysis of Perceptual Expertise in Radiology – Current Knowledge and a New Perspective

**DOI:** 10.3389/fnhum.2019.00213

**Published:** 2019-06-25

**Authors:** Stephen Waite, Arkadij Grigorian, Robert G. Alexander, Stephen L. Macknik, Marisa Carrasco, David J. Heeger, Susana Martinez-Conde

**Affiliations:** ^1^Department of Radiology, SUNY Downstate Medical Center, Brooklyn, NY, United States; ^2^Department of Ophthalmology, SUNY Downstate Medical Center, Brooklyn, NY, United States; ^3^Department of Neurology, SUNY Downstate Medical Center, Brooklyn, NY, United States; ^4^Department of Physiology/Pharmacology, SUNY Downstate Medical Center, Brooklyn, NY, United States; ^5^Department of Psychology and Center for Neural Science, New York University, New York, NY, United States

**Keywords:** visual perception, expertise, radiology, visual search, perceptual learning, attention, holistic processing, gist

## Abstract

Radiologists rely principally on visual inspection to detect, describe, and classify findings in medical images. As most interpretive errors in radiology are perceptual in nature, understanding the path to radiologic expertise during image analysis is essential to educate future generations of radiologists. We review the perceptual tasks and challenges in radiologic diagnosis, discuss models of radiologic image perception, consider the application of perceptual learning methods in medical training, and suggest a new approach to understanding perceptional expertise. Specific principled enhancements to educational practices in radiology promise to deepen perceptual expertise among radiologists with the goal of improving training and reducing medical error.

## Introduction

Optimizing perceptual expertise in radiology has great practical importance. One of the primary goals of radiology education is to train novices to develop advanced or ‘expert’ search methods to enhance abnormality recognition (Wood, 1999). The principles underlying radiologic expertise are also important beyond the immediate field as courts and policy makers rely on radiologists to provide testimony and educate juries on applicable standards of medical care (Andrew, 2006; Berlin et al., 2006). Despite continual efforts to refine radiology education, however, the error rate in radiological readings has not improved in the last seven decades (Garland, 1949; Berlin, 2007), persisting at a rate of approximately 33% for abnormal studies (Waite et al., 2017b). This problem—compounded by increasing imaging volumes and examination complexity—mandates a deeper understanding of the nature of radiological expertise, to improve both student training as well as the accuracy of practicing clinicians.

In this review we put forward that radiology’s error rate has been recalcitrant to improvement secondary to a lack of knowledge about the mechanisms of expertise. We further propose that no principled theories for improvement will be developed until we understand the precise nature of expertise in radiology. We discuss the definition of perceptual expertise in radiology, search strategies employed by experts, and current training methods. We also examine the limitations of extant eye tracking studies and visual processing theories regarding radiological expertise. Finally, we delineate a new approach to achieve a principled understanding of radiologic expertise, and how it will promote new heuristics (i.e., in designing individualized educational plans for each radiological trainee), with the ultimate goal of reducing radiological error (Gegenfurtner et al., 2017; Sheridan and Reingold, 2017; Waite et al., 2017b).

## What Does Radiologic Expertise Mean?

Radiologists are physicians who specialize in diagnosing and treating disease using a variety of medical imaging techniques such as x-rays, computed tomography (CT), magnetic resonance imaging (MRI), Positron emission tomography (PET), and ultrasound. In addition to a continually growing body of ‘fact-based’ knowledge regarding anatomy, radiological pathology, physics, and clinical medicine, expertise in radiology is considered largely perceptual in nature, defined by refined visual search patterns and diagnostic accuracy (Kelly et al., 2016). Thus, expert radiologists not only perceive abnormalities that non-experts do not, but they also better understand what to attend to and what to ignore (Gunderman and Patel, 2019).

Expertise in diagnostic imaging is usually inferred from the physician’s rank within the medical hierarchy—their title, level of training (i.e., intern, resident, attending, specialist), and years of experience—rather than by objective metrics. These indirect measures are presumably relied on because is difficult to accurately measure expertise. A principled understanding of perceptual expertise in specific tasks such as abnormality detection in radiology does not exist. If we could assess expertise with a biomarker instead —irrespective of physician’s rank—it would not only provide the basis to maximize accuracy and optimize heuristics, but it would also help determine the importance of training versus that of natural aptitude.

## Analysis of Medical Imaging

At a fundamental level, image analysis involves two basic processes: visual inspection of the image and interpretation (Krupinski, 2010). Broadly, diagnostic radiology entails (1) detection—noting a potentially significant finding is present that merits further analysis; (2) recognition—deciding that the finding is pathologic; (3) discrimination—characterizing the lesion as a specific type; and (4) diagnosis. The first task, detection, has primary importance, because all following steps leading to diagnosis rely on detection efficacy (Gray et al., 1978).

Perception is paramount in radiologic diagnosis because if a radiologist misses an abnormality, no amount of factual knowledge can remedy such a lapse; the diagnostic process may be prematurely terminated, resulting in misdiagnosis and subsequent harm to the patient. At the other end of the spectrum, false positives can also be detrimental to patient health. In both cases, until we better understand the nature of radiologic expertise, we will not be able to dissociate the contributions of perception and cognition to diagnostic accuracy.

## Error Rates in Radiology

Garland (1949) found that radiologists incurred an error rate of 33% in the interpretation of *positive* films (films that contain an abnormality), measured against the consensus of a group of experts. In a typical clinical practice (comprised of both normal and abnormal studies), the diagnostic error rate is approximately 4% (Siegle et al., 1998), a rate that translates into approximately 40 million interpretive errors per year worldwide (Bruno et al., 2015). Since Garland’s pioneering study, significant error rates have been noted in varied plain film modalities including mammography, chest X-rays (CXR), and bone X-rays, involving radiologists not only in private practice (Siegle et al., 1998), but also in academic settings, where interpretive error rates range from 13 to 90% depending on experimental conditions and the functional definition of error (Garland, 1949; Lehr et al., 1976; Forrest and Friedman, 1981; Muhm et al., 1983; Berlin, 2007; Brady, 2017). Recent studies of new technologies in radiology have determined that high error rates also exist in CT, MRI, and Ultrasound interpretation (Berlin, 2014; Herzog et al., 2017; Banaste et al., 2018).

Because of the subjective nature of radiologic interpretation, the definition of what is erroneous is established by expert opinion (Waite et al., 2017b). Thus, in a conclusive ‘error’ (as opposed to acceptable variation across observers), there is a substantial discrepancy with respect to peer consensus (Waite et al., 2017b). Although radiologic error can be classified in a number of ways (Kim and Mansfield, 2014), two broad categories of interpretive error are usually identified: perceptual errors and cognitive errors (Bruno et al., 2015). Cognitive errors are considered to occur when a correct positive finding is followed by misclassification due to faulty reasoning or lack of knowledge (Renfrew et al., 1992). Communication errors are an additional important cause of error, outside of interpretive or perceptual categorization (Waite et al., 2017c, 2018).

Omission or false negative errors occur when a radiologist fails to notice a perceptible lesion (as opposed to a fundamentally ambiguous lesion). A major source of diagnostic error and litigation, omission errors have been divided into three categories based on fixation times on missed lesions: search, recognition, and decision-making. Search errors are scanning errors in which the observer never fixates the lesion. Recognition errors are omission errors where the radiologist fixates the lesion for a duration shorter than the threshold dwell time (from 500 to 1000 ms depending on modality) considered necessary to recognize lesion features, and therefore fails to identify it. Decision-making errors are omission errors where the radiologist fixates the lesion for long enough to extract relevant lesion features, but dismisses the lesion as inconsequential (Kundel et al., 1978; Krupinski, 2010). Although only search and recognition errors are technically perceptual in etiology (Krupinski, 2010; Waite et al., 2017b), given the lack of eye tracking metrics during routine clinical imaging and most research studies, in practice (and in this review) all omission errors are usually termed ‘perceptual’ (Bruno et al., 2015).

Renfrew et al. (1992) classified 182 cases presented at a problem case conference and found that 43% were secondary to false negative or false positive readings. Kim and Mansfield analyzed 656 radiologic examinations with delayed diagnosis secondary to radiologic error and found that 42% were secondary to missed diagnosis (‘under-reading’) *without* an identifiable cause. An *additional* 42% were also secondary to missed diagnosis, but were felt to be attributable to a variety of causes, including satisfaction of search—where lesions remain undetected after the discovery of an initial lesion, alliterative error—where an error is made secondary to overreliance on a prior report, poor/misleading history, location of a finding outside the field of interest or at the corner of a film, and failure to consult prior radiologic studies/reports (Kim and Mansfield, 2014). In an analysis of 496 suits leading to malpractice claims, failure to diagnose was overwhelmingly the most common reason for initiating a malpractice suit against radiologists, comprising 78% of the cases (Baker et al., 2013). Funaki et al. (1997) and Rosenkrantz and Bansal (2016) found that missed findings accounted for 60–80% of interpretive error. Thus, faulty detection—failure to identify salient findings—is considered the most important source of interpretive error in radiology (Degnan et al., 2018).

Less discussed in the literature, false positive errors are also important to recognize. A major problem in screening examinations, false positive errors cause patient anxiety and often engender further unnecessary studies and procedures (Castells et al., 2016).

Given their ubiquity, interpretive errors are unlikely to be entirely due to bad radiologists (Brady, 2017). Indeed, given the high incidence of interpretive errors in essentially all radiologic scenarios—across multiple imaging modalities, and in both private practice and academic settings—a more probable explanation is that the methods to select potential radiology trainees, and resident education, are not better nowadays than 70 years ago.

## Attention and Perception

Voluntary attention—the selective processing of information at a given location—is deployed with specific targets in mind and guided to target locations by their prominent visual features (Wolfe et al., 1989; Wolfe, 1994; Alexander and Zelinsky, 2009, 2011; Carrasco, 2011, 2014; Eckstein, 2011; Anton-Erxleben and Carrasco, 2013; Carrasco and Barbot, 2014; Nobre et al., 2014). Whereas color, brightness, and motion are known to attract attention in a bottom–up manner (Wolfe et al., 2016; Wolfe and Horowitz, 2017), the observer’s intended target representation also directs attention in a top–down manner. Learning, memory, and expectations shape this process (Carrasco et al., 1998; Chen and Zelinsky, 2006; Wolfe et al., 2016). Thus, radiological search likely reflects a combination of bottom–up and top–down attention (Jampani et al., 2012; Wen et al., 2017).

By developing better target representations—for example, with increasing expertise in nodule detection—radiologists learn to remove many irrelevant areas from consideration, based on their understanding of the structure and content of radiological images (Wolfe et al., 2016). Thus, expert radiologists do not scrutinize all regions of the image equally but direct their attention and eye movements more precisely to relevant areas. This heightens efficiency but can also mask unexpected findings.

## Eye Movements and Search Patterns in Radiology

### Eye Movements and Expertise

The study of eye movements can reveal not only the cognitive processes behind expertise, but also the mechanisms involved in acquiring these skills. In addition, studies of expert gaze patterns can identify common perceptual errors leading to strategies to mitigate them– improving training and reducing error (Fox and Faulkner-Jones, 2017). As such, eye tracking technology has been increasingly used to understand the nature and acquisition of radiological expertise.

**FIGURE 1:**
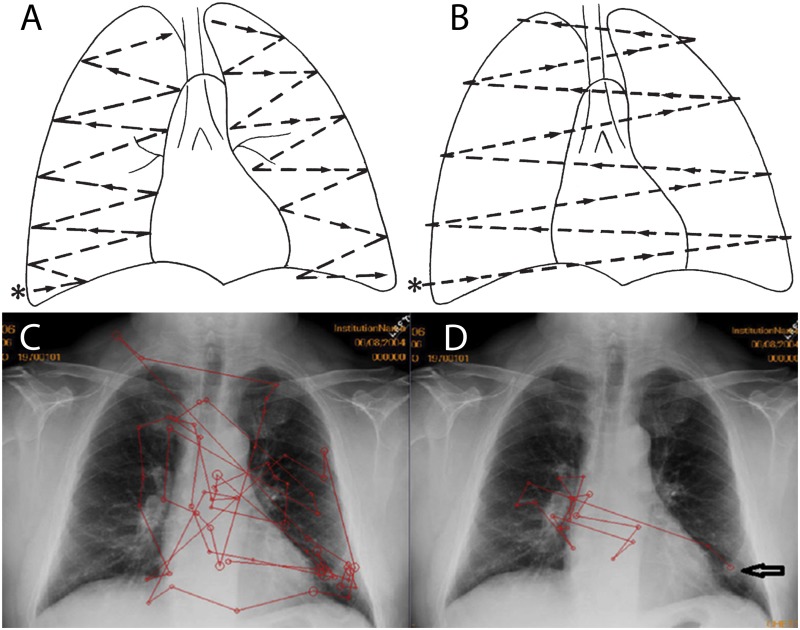
Reprinted from Goodman and Felson (2007) with permission. **(A,B)** Proposed lung search pattern. **(A)** This commonly taught search pattern for examination of the lungs during chest radiograph (CXR) interpretation involves starting at the right base (^∗^) (the costophrenic angle) and examining the right and then left lungs. **(B)** A second look is then performed in order to compare the right and left lungs as bilateral symmetry is assumed to be useful in recognizing abnormalities (Carmody et al., 1984). Reprinted from Waite et al. (2017a) with permission. Typical scanpaths of a novice **(C)** and an expert **(D)** radiologist, both searching a CXR which has a nodule at the left base (arrow in **D**). This free search pattern **(D)** is typically employed by experts and differs from the formal radiologic training given in residency. Instead, it indicates the flexible use of search strategies as a function of immediate visual information. The expert radiologist **(D)** has more efficient scanpaths (red lines) than the novice **(C)**, with fewer fixations (circles), less coverage of the image, fewer saccades, and faster arrival at the abnormality.

During interpretation, expert radiologists generally fixate on abnormalities faster than novices, and their total image search time decreases with increasing levels of expertise (van der Gijp et al., 2017a). Experts also have fewer total fixations than novices. These differences may be due to novices spending more time looking at irrelevant but salient structures [such as the heart on a chest x-ray (CXR), when the lungs are more important to analyze in a nodule detection task] with experts demonstrating more effective search secondary to refined scene guidance (Wolfe et al., 2016; van der Gijp et al., 2017a). Other gaze metrics, such as saccade length and image coverage, demonstrate less consistent experience-based differences across studies (van der Gijp et al., 2017a) (Figure 1).

Although most studies have examined the effects of expertise on plain film interpretation, expert radiologists are also more accurate and faster than novices during interpretation of *volumetric* imaging (Nakashima et al., 2016). In a study evaluating accuracy and interpretation in stroke CT and MRI cases, Cooper et al. (2009) found that attending radiologists were more accurate and had decreased time to fixation on lesions compared with novices. In addition, novices spent more time than experts examining normal anatomy, such as the ventricles (Cooper et al., 2009). Mallett et al. (2014) found that experts viewing a CT colonography video had shorter time to first pursuit on polyps (defined as the time from the beginning of video to fixation on the polyp for longer than 100 ms) compared with novices. A study of abdominal CT similarly found that attendings had higher accuracy than residents (Bertram et al., 2016). Another study found that experts had increased sensitivity toward medically important findings (such as nodules) compared with unimportant findings (such as bullae), suggesting specialization of their perceptual skills to detection of relevant findings (Nakashima et al., 2015).

Bertram et al. (2016) found that, whereas attendings and advanced residents performed better than less experienced residents when detecting low-contrast lesions on CT, detection of high-contrast lesions was comparable across groups. This finding suggests that expertise results in an increased ability to detect less salient abnormalities (Bertram et al., 2016).

### Relationship Between Fatigue, Expert Performance, and Eye Movements

Expertise may limit the effects of fatigue-related decreases in performance and changes in eye movements. Krupinski et al. (2012) found that residents had reduced detection accuracy in a CT nodule detection task after a day of reading, but the accuracy of attending radiologists was unaffected (Waite et al., 2017a). Similarly, Bertram et al. (2016) found that increased time at work resulted in decreased performance with CT scans for junior residents (with less than 2 years’ experience), suggesting that enhanced mental effort is more robust to fatigue in expert readers.

Hanna et al. (2018) found that after an overnight shift fatigued radiologists demonstrated worse diagnostic performance (with increased false negatives and positives) and increased time to fixate on fractures when examining bone radiographs. Although total viewing time per case was longer for all radiologists when fatigued, the effect was significantly more pronounced with residents compared to faculty members. In effect, in their fatigued states, faculty members had eye-tracking parameters more characteristic of non-fatigued residents (Hanna et al., 2018).

### Do High-Performing Radiologists Have Greater Preexisting Visuospatial Skills?

Given the possibility that ingrained aptitude could play a role in radiological success, measuring the perceptual abilities of radiology applicants and residents could be of great practical importance. The excellence of a group of professionals may be optimized through selection of individuals with the best requisite skill set. Radiology residency applicants are usually selected on the basis of academic records, letters of recommendation, and a short interview (Smith and Berbaum, 1991), none of which directly pertain to perceptual abilities. As Birchall notes, the existing model of training assumes that almost all trainees will eventually reach an acceptable standard with practice and semantic knowledge. Yet, it is possible that trainees with higher preexisting skills (i.e., in visual-spatial processing) may reach a *higher level* of expertise—or may achieve the highest level *more quickly*—than trainees with lower preexisting skills (Birchall, 2015). The identification of a relevant perceptual test might therefore help determine how much individual residents may benefit from training, and, ultimately, how they will perform as radiologists (Sunday et al., 2017).

The fact that radiology’s high rate of error has remained unchanged for over half a century may be partly explained by selecting applicants without regards to their perceptual (or ‘potential’ perceptual) abilities. It is unknown whether high-performing radiologists had greater baseline visuospatial skills at the start of their training or whether their ability was secondary to learned expertise (Corry, 2011). The persistence of high interpretive error rates and the occasional ‘spectacular failure’ suggest the necessity of reevaluating selection methods (Smith and Berbaum, 1991).

To this end, investigators have attempted to identify perceptual requirements for both learning and practicing radiology, as well as whether practicing radiologists have superior perceptual skills outside of imaging. Nodine and colleagues conducted a series of experiments comparing the performance of radiologists and laypeople in searching Where’s Waldo images from the popular children’s books (in which the challenge for readers is to find the character Waldo amongst a crowd of people). Radiologists and laypeople performed similarly, indicating that radiological expertise in visual search and/or perceptual discrimination did not carry over to non-radiological tasks (Nodine and Krupinski, 1998).

Bass and Chiles performed a series of visual and perceptual tests (including tests of visual acuity, contrast sensitivity, visual memory, visual completion, gestalt closure, identification of hidden figures in a picture, and three-dimensional construction ability) in medical students, residents, and faculty (Bass and Chiles, 1990). They found a correlation between performance on the hidden figures test and the ability to identify pulmonary nodules on a CXR for medical students, but not for residents or board-certified radiologists, suggesting that any innate abilities are quickly superseded by training (Bass and Chiles, 1990).

Similarly, Kelly and colleagues found no difference in performance or gaze dynamics across radiologists of varying levels of experience (ranging from interns to attendings) when asked to identify hospitals on a series of maps, or to find an anomalous shape within a group of similar shapes. These results reinforce the premise that radiologic expertise is a learned task-specific skill (Kelly et al., 2018).

Smoker et al. (1984) found a correlation between radiology residents’ ability to assemble Lego blocks by replicating a diagram and semiannual faculty ratings of their film reading performance. Because the results of the construction tests did not differ between residents and faculty with varied experience, the researchers concluded that the tests measured inherent aptitude rather than expertise (Smoker et al., 1984). Follow up studies confirming these preliminary results have not been performed, however, and no visuospatial ability test currently exists to determine whether someone is likely to become an ‘expert’ radiologist (Smith and Berbaum, 1991; Krupinski, 2011). Indeed, residency training programs do not objectively measure perception either before or during residency (Brazeau-Lamontagne et al., 2004). Unfortunately, taught declarative knowledge about *what* to look for does not ensure either expert perceptual abilities or act as a safeguard against ‘creative reading’ such as interpreting composite shadows as real nodules (Brazeau-Lamontagne et al., 2004).

A recent study by Sunday et al. (2017) found a modest correlation in naïve subjects between performance on the Vanderbilt Chest Radiograph Test (VCRT, where observers mark in which lung a nodule is when looking at two CXR’s) and on the Novel Object Memory Test (a domain-general test of novel object recognition). Further research may determine whether some individuals learn to recognize radiologic abnormalities faster than others, and whether preexisting abilities, such as measured by the VCRT, place a limit on one’s ultimate level of performance (Sunday et al., 2017).

### Search Pattern Instruction for Plain Film Imaging in Radiology

Research findings concerning human perception of medical images have not been widely translated into practical heuristics that improve training (Auffermann et al., 2016). Although there are published guidelines on how to interpret various radiologic examinations (Puddy and Hill, 2007; James and Kelly, 2013), they tend to be unprincipled and subjective, with few studies demonstrating their efficacy. When systematically analyzed, most of these educational tools have had mixed results.

Because novices lack the ability to generate a rapid and accurate global impression of an image, they may benefit from an orderly and comprehensive search pattern/order (Goodman and Felson, 2007; Auffermann et al., 2016) (Figure 1). If readers adhere to a specific order or search pattern in the inspection of anatomical structures, they may achieve more complete coverage of the image, reducing the number of overlooked abnormalities. Although full coverage could also be achieved by inspecting anatomical structures in a *random* order, keeping a specific order of inspection provides readers with a mental checklist (Kok et al., 2015). Thus, one commonly taught technique, “systematic viewing,” is to inspect anatomical areas in a fixed order.

Evidence to support the value of “systematic viewing” is wanting, however. Whereas, van Geel et al. (2017) found that students trained in systematic viewing methods inspected a larger portion of images than untrained students, they found no difference in their performance in chest radiographic interpretation. Kok et al. (2015) demonstrated similar findings. The combined data indicate that an emphasis on systematic viewing may not be justified.

Auffermann et al. (2016) found that physician assistants that were taught specific eye movements for analyzing CXR’s improved their ability to identify nodules and made less identification errors than those without such training. However, this study did not track the eye movements of participants. Thus, the effects of eye-movement training on search patterns or image coverage remain unknown (van Geel et al., 2017).

### Expert Search Patterns

One potential reason that pre-defined search patterns fail to consistently improve resident accuracy is that experts themselves do not read plain films in a consistent, standardized manner. Therefore, the various advocated search methods are not consistently used in practice (Kundel and La Follette, 1972) (Figure 1). Instead, experts use a variety of non-systematic search patterns, so-called ‘free search,’ when looking at images (Auffermann et al., 2016). An evolution of search patterns from medical students to attending radiologists was noted in one study but attending search patterns were not systematic; eye movements were more affected by the findings on the radiograph than any preplanned search pattern (Kundel and La Follette, 1972). A pioneering study by Tuddenham and Calvert (1961) found a wide variation in search patterns among readers. Ironically, the only observer with a reproducible search pattern failed to report findings noted by observers with inconsistent patterns (Tuddenham and Calvert, 1961). These findings are remarkably consistent with those from studies conducted over 50 years later, suggesting that consistent search patterns do not help, and indeed might be detrimental, to accurate diagnosis (Kok et al., 2015). Carmody et al. (1984) also found that, although radiologists are taught to compare both lungs (to look for asymmetric findings), less than 4% of their eye movements indicated such comparison scans, further illustrating the gap between radiologic instruction and practice.

### Search Patterns in Volumetric (3D) Imaging

**FIGURE 2:**
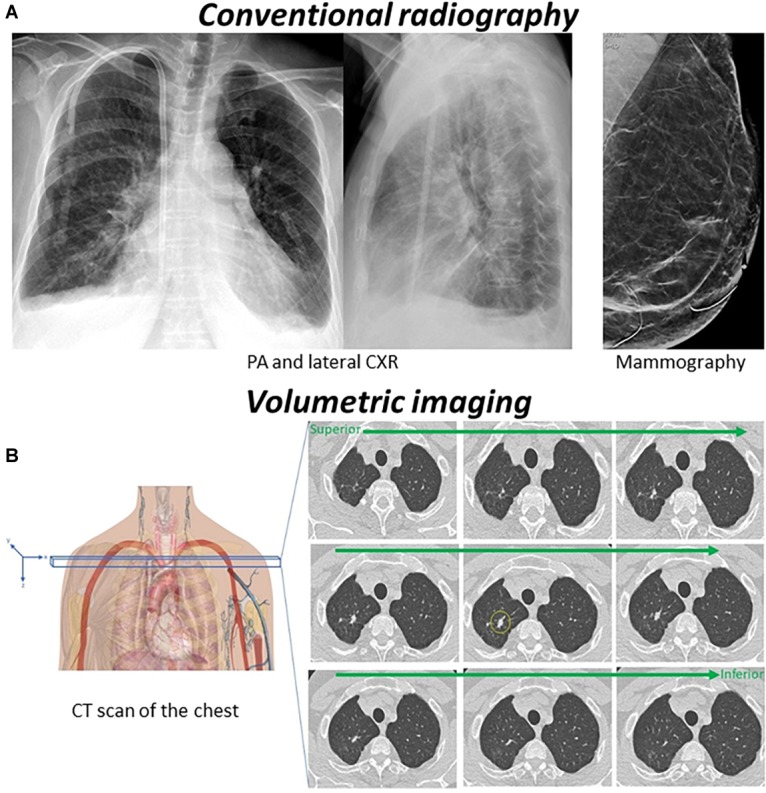
**(A)** Conventional radiography (2D medical imaging) such as CXR, mammography, and plain film bone X-rays, is based on the fact that tissue will absorb photons from an X-ray beam in relation to the electron density of the tissue. The number of photons passing through the region of interest will be detected by image detectors that convert the body’s direct attenuation of the photons into digital images. The resulting images are a two-dimensional projection of a three-dimensional structure. **(B)** In volumetric imaging such as CT and MRI, cross-sectional images of the body are obtained to represent a “slice” of the person being imaged, such as the slices in a loaf of bread. Once a number of successive slices are generated, they can be digitally “stacked” to form a three-dimensional image of the patient. On the CT scan above there is a nodule in the right upper lobe (yellow circle). As a radiologist scrolls through the stack, the nodule will present as a suddenly appearing (and then disappearing) lesion, simulating motion.

Modern medical imaging increasingly includes not only static image viewing (e.g., mammography and plain film radiography), but also dynamic imaging, such as with sequential viewing of multiple slices of CT and MRI, often in different orientations. When reading a CT or MRI, contemporary radiologists must scroll through a stack of images-thin slices of the 3-D volume of an organ–a process known as “stack viewing” (Nakashima et al., 2016) (Figure 2).

Although numerous studies have examined search strategies utilized in viewing single 2-D medical images, relatively little is known about those employed during interpretation of 3-D volumetric medical images (Nakashima et al., 2016).

In many ways, stack viewing is a different perceptual task than reading static images, and it may be regarded as a type of visual search conducted in a dynamic display. During stack viewing, radiologists search for the onset signal of a suddenly appearing lesion that stands out among blood vessels and organs that appear to move (simulated motion) (Nakashima et al., 2016). Thus, the fundamental characteristics found in the search of static images do not necessarily apply to the search of dynamic displays.

Drew and colleagues identified two different global strategies adopted by radiologists when searching through volumetric images—specifically during a nodule detection task in chest CT. “Scanners” searched each slice widely, before moving on to the next depth. “Drillers” held their eyes relatively still in the x and y plane, limiting their search to a single lung quadrant while quickly scrolling—drilling—through slices in depth. The data revealed a higher true positive rate for drillers than for scanners: drillers identified 60%, and scanners identified 48%, of all available nodules (Drew et al., 2013b).

Whereas drillers had more experience than scanners in the above dataset (Drew et al., 2013b), a follow-up study showed that instruction in drilling techniques led to improved performance in residents, compared with instruction in scanning techniques (van der Gijp et al., 2017b). These combined results support the superiority of drilling vs. scanning strategies. It is also worth noting that, in contrast with of Drew et al. (2013b) findings, none of the participants in the van der Gijp et al. (2017b) study reported (or had eye movements suggestive of) ‘scanning’ when reading images under normal work conditions. This is consistent with one of the authors’(SW) experience.

More recently, Kelahan et al. (2018) found evidence that the ‘drillers’ vs. ‘scanners’ model is likely imperfect when applied to CT scans of the abdomen and pelvis, given there are more organs in the abdomen and pelvis than in the thorax, and radiologists looked for multiple possible imaging findings (vs. only nodules in the Drew et al., 2013b experiments). It is likely that different search techniques are used depending on imaging modality and the part of the body imaged (e.g., thoracic vs. abdominal or neurologic imaging).

In order to get a more granular understanding of scroll behavior beyond the more general concepts of drilling and scanning (Drew et al., 2013b), Venjakob and Mello-Thoms (2016) and den Boer et al. (2018) defined different types of scroll behavior during CT interpretation. Quantification of the number of slice transitions performed before radiologists change their scrolling direction can provide insight into the desired image content (Venjakob and Mello-Thoms, 2016). den Boer et al. (2018) temporally related scroll movements to cognitive data via a think-aloud strategy, whereupon radiology residents verbalized their thoughts while reading CT scans. Half runs and oscillations (‘local’ movements covering less than 50% of the stack slices) were often associated with analysis [defined as cognitive activities including characterization of findings (van der Gijp et al., 2014)]. Runs (movements forward or backward covering more than 50% of slices) (Venjakob and Mello-Thoms, 2016) and image manipulation (where readers changed contrast levels or stack orientation) were more frequently associated with perception—search strategies and the global search for abnormalities (den Boer et al., 2018). Interruptions (where scrolling was paused) mainly coincided with synthesis related to the integration of information (e.g., generating a differential diagnosis) (den Boer et al., 2018). It is unclear whether these findings generalize to expert search or whether the relationship between scroll behavior and cognition changes with experience, however, studies of this kind can engender further insights into how radiologists interpret volumetric examinations.

Because of the large data inherent to volumetric imaging (often 1000s of images per study) (Andriole et al., 2011), radiologists do not exhaustively foveate all regions of interest, but rely on detection of signals with their peripheral vision (Eckstein et al., 2017). Drew et al. (2013b) found that radiologists covered, on average, 69% of the lung tissue on a CT nodule detection task. In a similar nodule detection task, Rubin et al. (2015) found that observers covered, on average, only 26.7% of the lung tissue. The difference between the results from these two studies could reflect their respective operational definitions of central field size (Kundel, 2015). Yet, despite their discrepancies, the main common finding in both studies is that a large amount of the lung tissue is never examined with foveal vision. Interestingly, Rubin et al. (2015) found that although observers foveated less than 33% of the lung volume, their search volumes encompassed an average of 75% of all nodules, showcasing the efficiency of peripheral detection (Kundel, 2015).

As peripheral vision cannot provide the kind of fine spatial discriminations that characterizes foveal vision, detectability of certain lesions can differ in 3D vs. 2D image searches (Eckstein et al., 2017). For instance, Eckstein et al. (2017) found higher detectability for calcifications in 2D single slice images—a Gaussian noise field tuned to be similar to the noise present in mammograms, and relatively improved detection of masses in 3D volumetric imaging– a stack of 2D images where the user could scroll up and down in the stack of images in a similar manner to the way a clinician explores a digital breast tomosynthesis case (Eckstein et al., 2017).

Models of saliency further illustrate differences between 2D and 3D search, predicting that different radiologic examinations are approached in distinct ways to optimize performance. Wen et al.,(2016) made a ‘dynamic saliency map’ where higher saliency is ascribed to motion flows that deviate from normal dominant flows, expected to reflect the observation that nodules pop out from anatomical backgrounds during volumetric interpretation. Alternatively, the Graph-Based Visual Saliency (GBVS) model predicts saliency due to the comparison of static image features within a given slice. Wen and colleagues demonstrated that driller fixations were aligned better with the dynamic saliency map and scanners with GBVS. This suggests that topographic maps representing conspicuity of objects and locations differ between radiological viewing methods, and more specifically, that scanners tend to use primarily 2-D information in their search, whereas drillers (the great majority of radiologists) use more dynamic information when interpreting cross-sectional imaging (Wen et al., 2016).

Both Wen’s and Eckstein’s studies lend support to the notion that 2D search and volumetric search are different perceptual tasks, and thus observer performance in one may not generalize to the other (Wen et al., 2016; Eckstein et al., 2017).

### Search Patterns and Spatial Frequency Analysis

The ability to detect a lesion is not only dependent on lesion characteristics, but also on the relation between the lesion and the background. As described in the holistic model of medical image perception, a target is compared to its background (which may camouflage the target, causing recognition error) to determine whether it is noteworthy (Nodine and Kundel, 1987). The relation between the lesion and the background therefore determines whether any given lesion will be above or below the detection threshold for a given decision criterion (Mello-Thoms et al., 2003). Mello-Thoms (2003, 2006), Mello-Thoms et al. (2003), and Mello-Thoms and Chapman (2004) performed a number of studies using image processing in order to understand the interplay between lesions and the parenchyma during mammographic interpretation. Using wavelet packet decomposition, they measured the log of the energy (energy being the integral of the signal strength) of different spatial frequency bands across a range of orientations and made a profile of measurements for each fixated region (Mello-Thoms, 2003). Because radiologists make comparative judgments between the background and any potential lesions, Mello-Thoms et al. (2003) considered not just the profile in each region (termed the local profile) but also a combined profile (the global profile) across all fixated regions in an image. The local profile is related to the conspicuity of local features and the global profile is a measure, specific to each observer, of the searched background to which local features are compared (Taylor, 2007).

Both residents and mammographers were visually attracted to similar areas of mammograms, despite mammographers having significantly superior accuracy (Mello-Thoms et al., 2003). The data showed that the computed profiles did not discriminate between mammographers’ true- and false-positive decisions, suggesting that they possess mental schema (well-modeled by the spatial frequency representation) about how malignant lesions should look, and they do not deviate from that schema even when they make a mistake (a false positive). The profiles did, however, discriminate between mammographer’s true-positive and false-negative decision outcomes. For residents, however, there were statistical differences between false and true positives, and the global profile seemed to play a much smaller role in their decision-making, implying that conspicuity of local elements is strongly related to their decisions. Residents were less able to contrast local findings with global features, which misguided their judgments (Mello-Thoms, 2003; Mello-Thoms et al., 2003). Further understanding of what imaging features attract visual attention in conjunction with decision making can afford more specific training- for example by concentrating on residents’ improving their comparison skills during training (Mello-Thoms, 2003).

## Challenges to Eye Tracking Studies

Simple eye movement measurements may only provide meaningful data on radiologic expertise in certain imaging scenarios (Jarodzka and Boshuizen, 2017). For example, time to first fixation and the proportion of time spent on relevant findings, is only informative in the case of localized diseases, but not for diseases that are diffuse in nature (and may thus require more complex metrics) (Kok, 2012).

Kok (2012) found that radiologists examine diffuse and focal diseases on chest X-rays differently. In addition to ‘standard’ eye tracking measures they examined the ‘global/local ratio’ of saccades—computed by dividing the number of ‘long’ by ‘short’ saccades (respectively defined as greater and less than 1.6 degrees of visual angle). A higher ratio indicated a global, dispersed viewing pattern, and a lower ratio indicated local clusters of fixations in specific regions (Kok, 2012). Although there were significant differences in interpretation accuracy (determining the *likely* diagnosis for the examination) between attending radiologists and medical students (no difference was found in accuracy between residents and attendings), the global/local ratio between groups was similar in diseased images. The fact that all groups changed their viewing pattern according to the type of disease, but that medical students had significantly worse interpretation accuracy, is evidence that perceptual aspects of image interpretation- *detecting* abnormalities- develop before the ability to correctly interpret abnormalities or integrate them into a correct diagnosis (Kok, 2012). Further studies with more complex oculomotor metrics will be needed to unravel the relationship between eye movements and diagnostic expertise.

A recent study by Kasprowski et al. (2018) challenges the presumed relationship between visual search efficiency and diagnostic accuracy. They analyzed various eye movement metrics while four participant groups viewed CXRs preceded by proposed answers: observers with no medical experience, radiology technicians (‘radiographers’- skilled in performing X-ray examinations but not involved in diagnosis), radiology residents, and radiology attendings. As expected, attendings had the highest average diagnostic accuracy, followed by residents, radiographers, and laypeople. Interestingly, although there were significant differences in the eye movement metrics of residents and attendings, there were no significant differences between the visual patterns of radiology *technicians* and attendings, despite residents being significantly more accurate. The authors surmise that this finding may reflect general experience analyzing X-rays. Their participant radiographers had over 10 years’ experience checking the *technical* quality of imaging, compared to participant residents, who had less than 6 years’ experience, albeit for a different purpose—diagnosis. Thus, the ability to reproduce characteristics of experts eye movements does not guarantee improved diagnostic performance (Kasprowski et al., 2018). Expertise in radiology requires not only efficient, task-oriented, eye movements but accurate recognition of abnormalities.

An additional confound is that eye tracking metrics are not only dependent on imaging findings and reader expertise, but are also influenced by clinical history (reviewed in Waite et al., 2017b) and reader expectations of abnormal findings. In an analysis of 16 studies comparing the accuracy of tests with and without clinical information, Loy and Irwig found that clinical information improves interpretive accuracy through improved sensitivity without a loss of specificity, consistent with readers being alerted to additional imaging features, rather than by merely altering their level of suspicion (Loy and Irwig, 2004). Although many of these studies were not performed with associated eye tracking metrics, search patterns likely also vary in conjunction with changes in accuracy. Moreover prevalence expectation changes search patterns. Reed and colleagues studied performance during a CXR-based nodule detection task under conditions where readers were told that the images contained a specific number of abnormal findings. Higher prevalence expectations were linked to increased reader fixations and total analysis time (Reed et al., 2011). A follow-up study demonstrated that this ‘prevalence effect’ was particularly evident in normal examinations, with readers exhibiting decreased confidence that normal images were in fact normal (Reed et al., 2014).

A more fundamental challenge to assessing the findings of many eye tracking studies’ relates to their experimental design. Gur et al. (2008) and Gur (2009) found that radiologic performance during the interpretation of mammograms was different in real-life vs. laboratory settings.

Lastly, Van der Gijp and colleagues noted that studies conducted over the last two decades have largely focused on the differences between experts and novices, while theory-driven research on how to improve detection has been relatively neglected. Thus, there is a need for the field to move beyond the description of differences between experts and novices, to the development of more efficient strategies and methods to accelerate and improve training (van der Gijp et al., 2017a).

## Holistic ‘Gist’ Processing Theory

Prevailing models of medical image perception rest on the premise that expert observers process a medical image holistically at the first glimpse. These holistic processing accounts are encompassed by a couple of different theoretical frameworks, the oldest described being the global-focal search or ‘holistic’ model (Nodine and Kundel, 1987; Litchfield and Donovan, 2016; Sheridan and Reingold, 2017).

**FIGURE 3:**
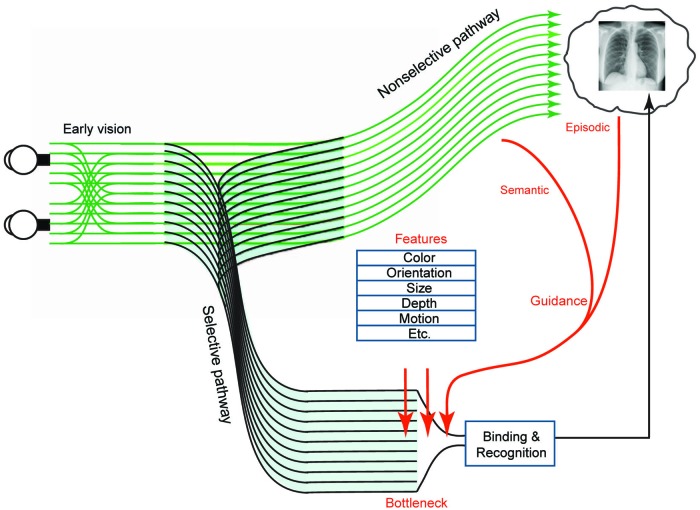
Figure modified from Wolfe et al. (2011) with permission. Two-pathway architecture for visual processing. The selective pathway can bind features and recognize objects but is capacity-limited. At its bottleneck, preference for further processing is given to items with certain basic attributes (such as color, orientation, and size) when those attributes match the appearance of a target object. However, these attributes do not fully explain the efficiency of search in the real world, where elements are arranged in a rule-governed manner—for example, people generally appear on horizontal surfaces. The regularity of scenes provides two kinds of scene-based guidance—semantic guidance, referring to the knowledge of the probability of the presence of an object in a scene and its probable location, and episodic guidance, referring to the memory of a *specific* previously encountered scene. In conjunction with the selective pathway, the non-selective pathway extracts statistics such as velocity, direction of motion, and size, rapidly from the entire image. Although the non-selective pathway does not support precise object recognition, it provides information used in scene-based guidance to direct attention to important locations (such as the probable locations of nodules on CXR’s). Conscious experience of the visual world is comprised of the products of both pathways (Wolfe et al., 2011).

According to the global-focal search model, medical experts rapidly extract a global impression of an image. This impression consists of a comparison between the contents of the image and the expert’s prior knowledge about the appearance of normal and abnormal medical images (i.e., the expert’s schemata). This enables experts to identify perturbations (deviations from their schemata that indicate possible abnormalities) and direct their eyes toward their corresponding locations for further (i.e., foveal) examination (Nodine and Kundel, 1987; Sheridan and Reingold, 2017). Features are subsequently scrutinized and tested against schemata to determine whether a finding is suspicious, in which case diagnostic decisions are made (Waite et al., 2017b) (for a more complete review see, Kundel et al., 2008). This process, lasting seconds to minutes, is capacity-limited by the bottleneck of attention (Waite et al., 2017b). Similar time constraints apply to all models that rely on global processing as a core component of expertise in medical image perception (Sheridan and Reingold, 2017). Another popular model posits that initial global processing (consisting of bottom–up “global image statistics” like average orientation and average size of objects) signals if there is an abnormality (establishing its likelihood) without providing *location* information or constraining the subsequent serial search. The searcher can then change their strategy to a slower, more complete search for the abnormality (Evans et al., 2010, 2016; Drew et al., 2013a; Chin et al., 2018) (Figure 3).

One of the global-focal model’s principal predictions is that rapid initial global processing constrains search to suspicious areas in the image (Carrigan et al., 2018). This strategy may be available to experts but not novices, explaining why expert observers search medical images with higher efficiency—finding more abnormalities in a shorter timeframe, and with fewer eye movements—than novices do. Support for this hypothesis has been provided by studies showing that expert radiologists can identify subtle abnormalities on mammography and chest radiography displayed for only 250 ms (Kundel and Nodine, 1975; Oestmann et al., 1988; Krupinski, 2011; Evans et al., 2013; Sheridan and Reingold, 2017; Carrigan et al., 2018).

Using time to first fixation on lesion data during mammographic interpretation, Kundel and colleagues found that 67% of cancers were fixated on within the 1^st^ second of viewing. The remainder of the cancer locations were fixated on later in search. This has been interpreted as further supporting a two-component system- rapid initial holistic processing guides initial search with subsequent slower processing representing discovery of cancers from search and discovery (Kundel et al., 2008).

The idea that holistic processing is integral to expert performance is also supported by experiments designed to disrupt it. Oestmann et al. (1993) found decreased detection performance for subtle lung cancers in upside-down images, even with unlimited viewing times. In a related study, Carmody et al. (1980) compared nodule detection performance in two viewing conditions: *segmented search*—in which the CXR was presented in six sections and viewed piecemeal—versus *global search*—in which the entire film was presented—and found an increased false positive rate in the segmented search scenario, which they attributed to an impaired gestalt.

**FIGURE 4:**
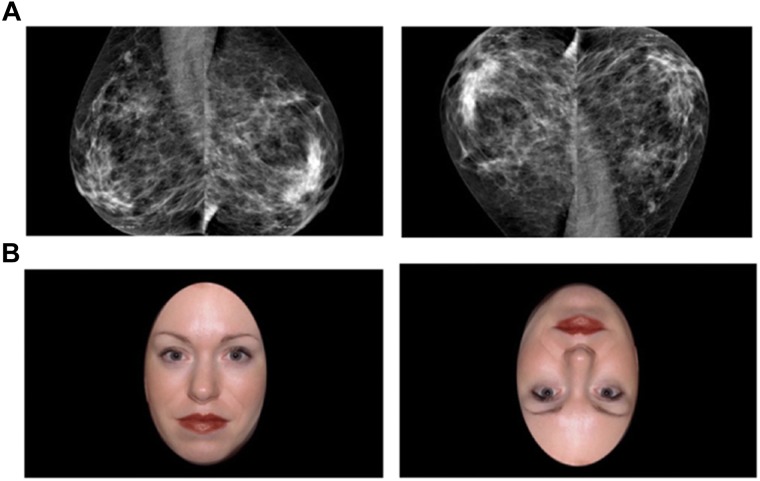
Reprinted from Chin et al. (2018) with permission. Examples of mammogram and face stimuli in upright and inverted orientations. **(A)** Upright and inverted abnormal mammogram. **(B)** Upright and inverted smiling face. The inversion effect posits that stimuli are processed as an integrated whole rather than a sum of its parts; therefore, the inverted image is harder to recognize and process (Chin et al., 2018).

Face perception is considered a prime example of holistic processing, where recognition is based on the synthesis of facial features that yields a unique face more than the summed recognition of each individual facial feature. The ‘gold standard’ for testing holistic face processes is the face inversion task, whereupon inversion *disproportionately* impairs the recognition of faces relative to other object classes. Turning a face upside down is thought to disrupt normal holistic face processing, forcing participants to use a less optimal strategy based on analysis of specific features (e.g., wide-set eyes, square jaw). Processing in mammographic interpretation appears to share similar characteristics to this holistic perception. In a recent study, Chin et al. (2018) tested the effects of image inversion during interpretation of normal and abnormal mammograms-inverted and normally oriented-by experienced radiologists and radiology residents (Figure 4). Participants were also asked to judge the facial expressions (e.g., neutral/happy) of briefly presented upright and inverted faces (Chin et al., 2018). Both groups demonstrated better expression discrimination of faces in the upright compared to the inverted orientations, as expected. However, detection rates for upright and inverted mammograms depended on expertise level. Whereas radiology residents were unaffected by image orientation, experienced radiologists performed better on upright images than on inverted ones. In addition, although accuracy in the upright position increased with years of experience, the magnitude of the inversion effect *also* increased, demonstrating that use of holistic strategies increases as a function of domain-specific perceptual experience. In short, expert holistic processing helped in detection of an upright stimulus but provided no advantage when the image was inverted (Chin et al., 2018).

A recent study by Brennan et al. (2018) suggests an intriguing relationship between expertise and gist processing. They found that not only could radiologists detect cancer above chance in abnormal mammograms viewed for only ½ second, but they were able to differentiate between normal mammograms in patients that *remained cancer free after 2 years* from normal mammograms in patients that subsequently developed cancer. These results suggests that mammograms without overt signs of cancer can contain information that may predict future malignancy. The readers that most rapidly differentiated between positive and negative mammograms were also better at differentiating between normal mammograms in patients that remained cancer free and normal mammograms in patients where cancer developed later. Their results suggest that expertise may increase radiologists’ capacity to perceive the ‘gist of the abnormal,’ an ability to detect a globally elevated risk of cancer in studies without overt radiographic signs (Brennan et al., 2018).

In a related experiment, Evans and colleagues found that expert mammographers demonstrated above-chance classification of a study as abnormal when shown 500 ms images from the *normal breast* in patients with overt signs of cancer in the *opposite breast*. This suggests that a widely distributed ‘global signal of abnormality’ was present in the normal breast parenchyma (Evans et al., 2016). Evans et al. proposed that such a signal, if present before the appearance of a clinical lesion, could be used as a warning sign suggesting greater vigilance (Evans et al., 2016). One possible way to accomplish this would be to flash an image for a half-second, record the readers’ gist response *before* usual presentation, and then use this signal to predict future breast cancer (Brennan et al., 2018). In conjunction, given a high gist response correlates with cancer in the *current* image, it might be cost-effective to send an image to a second reader for double reading when a case is classified as abnormal from the gist response, but reported as *normal* after usual presentation (Brennan et al., 2018).

## Challenges to Holistic Processing Theory

### Volumetric Imaging

Holistic processing theory is at best incomplete as a model of perceptual expertise in the era of modern imaging. The studies that buttressed the holistic processing model were conducted with plain 2-D imaging such as CXR (Kundel and Nodine, 1975; Oestmann et al., 1988) and mammography (Evans et al., 2013), but the nature of 3D volumetric imaging (with the necessity to scroll through images in the z-plane to detect abnormalities) is such that no single image can provide meaningful global image statistics or afford knowledge of image perturbations throughout the entire dataset. Therefore, there is no rapid ‘global signal’ that can be extracted from any single image to either organize subsequent fixations or contribute to the reader’s conviction that a subsequent search will uncover an abnormality.

### Flash Preview Moving Window (FPMW) Experiments

One of the problems with assessing the validity of the holistic model in radiology is that the corresponding studies were conducted under free-viewing conditions. Because observers had constant access to the whole scene via peripheral vision, it is difficult to isolate the specific contribution of the initial scene preview on subsequent eye movement behavior (Litchfield and Donovan, 2016).

**FIGURE 5:**
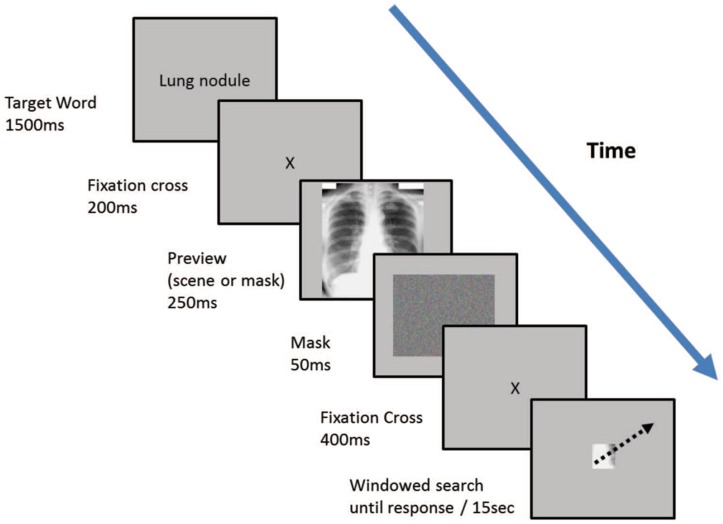
Reprinted from Litchfield and Donovan (2016) with permission. Two different experience groups—expert radiologists and psychology students—searched for lung nodules from CXR images using the flash-preview moving window (FPMW) paradigm. Participants looked at the target word for 15 s (“lung nodule”). They then saw a fixation cross for 200 ms, then either a mask preview (random array of colored pixels) or a CXR (a ‘scene’ preview) for 250 ms. Next, participants saw a mask for 50 ms and then a second fixation cross for 400 ms. Following a second presentation of the fixation cross, they conducted a windowed search, with a 2.5-degree radius window restricting the field of view (Litchfield and Donovan, 2016).

The flash preview moving window (FPMW) protocol draws from both ‘flash’ methodology and a ‘moving window’ paradigm. A brief preview of a scene is shown to observers, who must then search for a target within the scene while their peripheral vision is restricted (Litchfield and Donovan, 2016). To control how much of the scene is accessible for visual processing, a mask of variable size is tied to the observer’s central fixation, occluding the rest of the scene outside the fixation window. In this way, observers remain free to make eye movements, while researchers systematically control how much foveal, parafoveal, and peripheral information is available for visual processing. As a result, observers may examine a scene solely with high-resolution foveal vision, isolated from parafoveal or peripheral contributions (Litchfield and Donovan, 2017) (Figure 5).

Using this protocol, Litchfield and colleagues found that, whereas experts (consultant radiologists) identified more nodules on CXR than novices (psychology undergraduates), denying experts an initial glimpse of the image did not impact their performance. In contrast, novices performed better in the *mask* preview condition than with the scene preview. Further, the first eye movements following the scene preview and the mask preview had comparable speeds and amplitudes (Litchfield and Donovan, 2016). Thus, contrary to predictions from the holistic processing theory, the provision of an initial glimpse of the scene did not contribute to expert performance—and indeed *reduced* novice performance.

A follow-up experiment compared the detection performance of novices and experienced radiographers in three different imaging modalities—CT scan of the head, skeletal x-rays, and CXRs—for specific target abnormalities. As expected, the detection skills of experienced radiographers surpassed those of novices. However, access to a scene preview not only did not benefit, but actually *impaired* the performance of observers in both groups (Litchfield and Donovan, 2016). These combined findings argue against the hypothesis that processing the initial glimpse of a scene is beneficial to performance (Litchfield and Donovan, 2017) (but see Sheridan and Reingold, 2017 for a counterpoint).

### Scene Processing Without Attention?

We also note that flash preview experiments (Kundel and Nodine, 1975; Krupinski, 2011; Evans et al., 2013) are thought to support the holistic processing hypothesis based on the assumption that searchers only use information available *while* images are displayed (Sheridan and Reingold, 2017). Yet, there is reason to believe that image processing can continue *after* images are no longer displayed, even in the presence of a mask (Carrasco and McElree, 2001; Carrasco et al., 2003).

More generally, gist processing is thought of as a type of scene processing that occurs in the absence or near absence of attention (Li et al., 2002; Rousselet et al., 2002; Fei-Fei et al., 2005; Otsuka and Kawaguchi, 2007), in parallel across the visual field (Rousselet et al., 2002). But even if the initial glimpse of a scene is demonstrably helpful, it does not necessarily follow that searchers process information without attention. Instead, it may be that searchers rapidly extract visual information during a single brief fixation that provides high-resolution information at the fovea, and lower-resolution information in the visual periphery (Livingstone and Hubel, 1988; Strasburger et al., 2011), facilitated by attention. Attentional allocation to the periphery might involve one or more serial shifts of selective processing, either covertly during the first fixation or while continuing to process the image in memory, before the observer is required to provide a response. Carrasco et al. (2003, 2004, 2006) showed that peripheral stimuli continues to be processed after being removed from view, with the temporal dynamics depending on where stimuli are in the visual field, within the timeframe allowed by many gist experiments. Either scenario would be contrary to the concept of gist as occurring merely in parallel across the visual field and in the absence or near absence of attention.

## The Development of Perceptual Expertise in Radiology

Kelly and colleagues found that certain ocular metrics (such as time to first fixation) improved at an earlier stage of training than diagnostic accuracy in pneumothorax detection. This same study found significant differences in diagnostic accuracy, but not in the ocular metrics, of consultants (equivalent USA rank: attending) vs. registrars (equivalent USA rank: fellow), suggesting that expert gaze dynamics are learned at a faster pace than diagnostic abilities, and that they plateau at a relatively early stage of formal residency training (Kelly et al., 2016).

**FIGURE 6:**
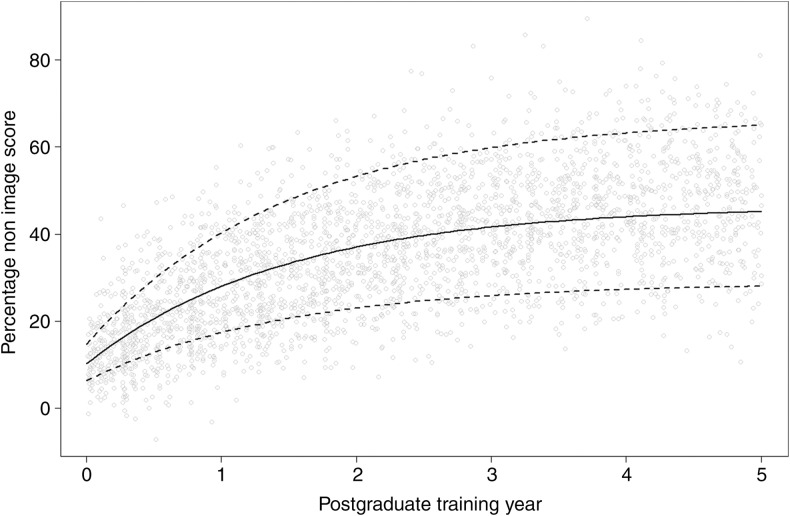
Reprinted from Ravesloot et al. (2017) with permission. Graph estimating image interpretation skill development during residency, as measured by the Dutch Radiology Progress test. Image score measures performance on image interpretation skills as opposed to factual knowledge. The score represents percentage from the maximum possible score and is calculated by subtracting the number of incorrect answers from the number of correct answers to account for guessing (making a negative value possible). The slope represents the speed of skill development and measures 16.8% during the first year of training. The slope decreases by 50% every year until it reaches 2.0% at the end of training. Note that the maximum image-score is estimated at 55.8%. Dotted lines represent the middle 95% of performances (Ravesloot et al., 2017).

Ravesloot et al. (2017) found that scores from image interpretation questions (image-based questions testing interpretation skills) improved faster than knowledge-based questions (text-based factual questions) for the first 3 years of residency when residents took the Dutch Radiology Progress Test, a mandatory semiannual test taken by all Dutch radiology residents (Figure 6).

Both eye tracking metrics and performance on image interpretation-based questions show that the ability to analyze images develops at a faster rate than factual knowledge. Whereas radiologists’ perceptual skills begins to grow from the start of exposure to imaging, radiology-specific factual knowledge contributes little to this initial development (Ravesloot et al., 2017).

Together, these studies also indicate that perceptual expertise plateaus early with a high level of error. Worse, this plateau likely persists beyond a radiologists formal training period, an important issue for residency programs to address.

## Challenges to the Functional Definition of Expertise

The functional definition of expertise in the literature is limited for a number of reasons. Radiologic learners are often classified into the broad categories of experts versus novices, dramatically oversimplifying reality while disregarding intermediate training stages (Gunderman et al., 2001). Even studies that include intermediate stages in their analyses base their categorization on the *professional* level of training (Kundel et al., 2007). Indeed, a large meta-analysis of eye tracking research in professional domains found that in 6 of 8 radiology-based studies, expertise was determined solely according to professional levels of training and/or years of experience (Gegenfurtner et al., 2011). Fox and Faulkner-Jones (2017) note that experimental designs tend to include at most three participants groups, and bin medical students as novices, radiology residents as intermediate-level trainees, and attendings as experts.

The assignment of the ‘expert’ label purely based on professional degrees, rank, or experience is problematic for reasons both theoretical and practical. Although a specific attending may be considered to be an ‘expert’ by peers because of an extensive knowledge base, this does not necessarily directly translate into perceptual expertise. As Gunderman notes, expertise in one radiologic domain does not necessarily apply to every domain (Gunderman et al., 2001). For instance, Beam et al. (2006) and Elmore et al. (2016) note that mammographers have two distinct tasks: (1) Interpretation of screening mammograms and (2) Interpretation of abnormal screening mammographic findings. The first task requires evaluation of standard images from a large population of individuals without specific signs or symptoms (considered ‘perceptual’), whereas the second task requires careful analysis of specific abnormalities (considered more ‘cognitive’) (Beam et al., 2006; Elmore et al., 2016). Both studies found only a *moderate* correlation between radiologists’ performance in the two domains. In other words, proficiency in one area did not guarantee proficiency in the other (Beam et al., 2006; Elmore et al., 2016). The presence of wide disparities in screening and diagnostic interpretive skills in the same individual has been described as ‘expertise disequilibrium’ (Beam et al., 2006). In addition, assuming perceptual expertise based purely on experience negates any potential effects from age-related declines in contrast sensitivity, known to be most marked for high spatial frequencies (Owsley and Burton, 1991; Davies et al., 1994).

It follows that, even if attendings are considered ‘experts’ by their peers (or by rank or by years of training), they are not *all* de-facto *perceptual* experts and indeed may be highly capable in only a subclass of the complete radiology skillset. This heterogeneity is problematic if, in fact, homogeneity of expertise is assumed when evaluating radiologists’ perceptual performance (i.e., such as in a nodule detection task study).

Bearing out these theoretical concerns, several research studies have demonstrated that residents in the ‘intermediate bin’ can outperform ‘expert’ attendings. Rubin et al. (2015) found no significant correlation between experience and detection of artificially placed nodules in CT examinations. Indeed, at least one resident in their *first* year of training outperformed a *sub-specialist thoracic radiologist* in their specialty, despite an extensive experience differential (Rubin et al., 2015). Similarly, Kelly et al. (2016) found that less experienced radiologists occasionally outperformed more experienced radiologists in pneumothorax detection. Kundel et al. (2007) found a wide range of diagnostic performance across residents, fellows, and attendings when looking at a test set of mammograms. Hanna et al. (2018) likewise found that residents could outperform attendings in a fracture detection task under both fatigued and non-fatigued conditions. Lesgold et al. (1988) discovered that more advanced residents were occasionally less likely to make a correct interpretation on several complex x-rays compared to more junior residents. Finally, Nakashima et al. (2016) found no difference in performance in a nodule and bullae detection task on CT amongst radiologists with experience ranging from 3 to 15 years.

In summary, experience is a necessary, but insufficient, indicator of expert performance (van der Gijp et al., 2017a). At best, experience is an uncertain predictor of expertise level, and at worse, it reflects little more than seniority (Shanteau et al., 2002). Likewise, the use of certification (such as conferred from the American Board of Radiology) as a marker of expertise is limited in that it is most often tied to years on the job, rather than to objective performance. To compound the problem, certification suffers from the ‘ratchet up effect,’ whereupon individuals move up but not down the ladder, even if their skill level suffers a serious decline over time (Shanteau et al., 2002). Ericsson has noted that there is little empirical evidence for the traditional view that expertise is acquired through extended experience alone (Ericsson, 2004). Instead, the development of expertise more likely arises from domain-specific ‘deliberate practice,’ with accurate and detailed immediate feedback and opportunities for repetition (Causer et al., 2014; Ericsson, 2017). It follows that if a first year resident outperforms a fellowship-trained thoracic radiologist in nodule detection, the resident should be labeled as an ‘expert’ (in studies of *search* tasks), irrespective of their lack of experience. Defining expertise by performance, rather than by titles, is therefore liable to produce more accurate data.

## A Way Forward—Ways to Promote Perceptual Expertise

### The Role of Subspecialty Training

Given increasing specialization in medicine, some authors have advocated that radiology groups pursue a subspecialization model (Strax, 2012; Gunderman and Stevens, 2014; Arenson, 2018). Indeed, several studies have shown that subspecialists have better accuracy in their relative subfields than general radiologists (Sickles et al., 2002; Briggs et al., 2008; Bell and Patel, 2014; Kligerman et al., 2018), perhaps due to limiting the field of scope and enhanced networking with subspecialty medical experts whom provide prompt feedback. Thus, concentration on a narrow field may be a way to achieve the case volume and feedback needed to ensure expertise.

### Perceptual Learning

Perceptual learning may be defined as “an increase in the ability to extract information from the environment, as a result of experience and practice with stimulation coming from it” (Gibson, 1969). In general terms, this refers to how experience can change the way we perceive sights, sounds, smells, tastes, and touch. Such continuous learning is the foundation of perceptual competence and eventual expertise. As such, it is a topic of intense and increasing scientific study. For recent reviews see (Lu et al., 2011; Gilbert and Li, 2012; Sasaki et al., 2012; Kawato et al., 2014; Li, 2016; Seitz, 2017).

**FIGURE 7:**
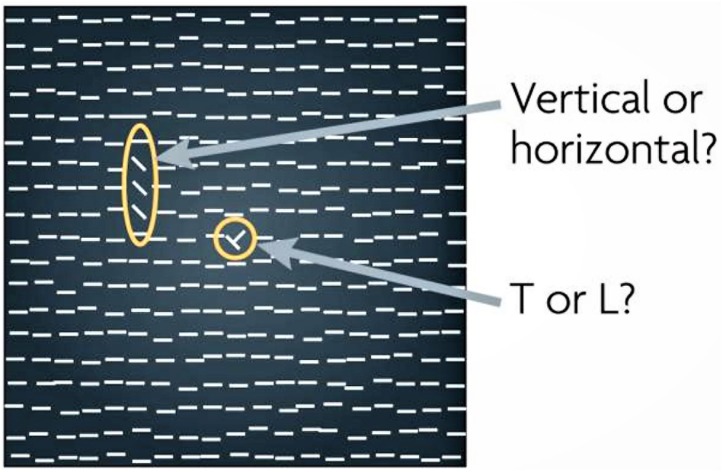
Reprinted from Sasaki et al. (2010) with permission. Texture-discrimination tasks such as depicted here are frequently used in visual perceptual learning (VPL) studies. The subject is first asked to report whether a ‘T’ (as in this example) or an ‘L’ is presented in the center of the display to ensure fixation, and then whether the orientation of the target (which is comprised of the three elements with orientations that depart from those of the other elements in the display) is vertical (as in this example) or horizontal. VPL of the target orientation is examined.

The developing brain calibrates its perceptual systems through interaction with the environment; for instance, tuning and updating neural representations of our body and sensory organs as we grow (i.e., the length of our limbs changes with age). Much of this learning occurs during critical periods in early infancy, when the brain is most plastic, and its processes and connections are easily molded by experience. It is during such critical periods that changes in visual experience can have profound impact on the functional organization of the brain regions underlying perception. Yet, even though neural plasticity is diminished in adults, it is not lost. Indeed, with proper training, adults can exhibit an impressive degree of perceptual learning. A variety of stimuli including texture, orientation, contrast, and motion are used in visual perceptual learning research (Figure 7).

Perceptual learning studies most often focus on a specific visual feature, such as orientation, and train participants with exemplars. Typically, performance thresholds improve over time, so that by the end of training participants can accurately discriminate stimuli that would have been indistinguishable to them at the study’s onset. This type of learning differs from more conventional instruction methods where training is understood as consisting of facts, concepts, and procedures that are stored in one’s mind and later retrieve as needed for performance. This “container” model of the mind has come into question, however, in light of persistent problems in learning and instruction (Kellman, 2013). Students who have been faithfully taught and diligently absorbed declarative and procedural inputs occasionally fail to recognize key structures and patterns in real-world tasks. In addition, they may know how to perform procedures but fail to understand their application, especially to new problems or situations. Lastly, learners may understand concepts but process them slowly, with a high cognitive load, which leads to impairment in demanding, complex, or time-limited tasks (Kellman, 2013). One of us (SW) has encountered medical students who, after reviewing basic CXR principles from textbooks for a month, did not know how to apply their declarative knowledge in any practical sense, to interpret novel examinations. In other words, students may be able to memorize isolated, inert facts, but lack the ability to use them constructively to solve problems in radiologic diagnosis (Gunderman et al., 2001).

Whereas novice trainees tend to engage in more feature-based ‘analytical’ processes, experts perform more rapid pattern recognition, which relies in part on experience-based perceptual (a form of ‘implicit’) learning (Rimoin et al., 2015). Two broad categories of perceptual learning effects have been described: discovery and fluency (Kellman et al., 2008; Kellman, 2013). Discovery effects involve finding information relevant to classifications, extracting it selectively and distinguishing important structures from irrelevant variations. Fluency effects involve improvements in the extraction and encoding of relevant information. Effects include speed, parallel processing, and automaticity (experts demonstrate lower cognitive load than novices), allowing efficient perceptual classification to coexist with other cognitive processes in complex tasks (Krasne et al., 2013).

One of the major problems in radiology education is the lack of formalization and verbalization of what exactly happens during visual information extraction (i.e., “How do you teach to *see* a nodule?”) (Kellman and Garrigan, 2009; Krasne et al., 2013). Instead, it is assumed that advanced pattern recognition and automaticity will eventually arise from long apprenticeships, leaving crucial aspects of learning to occur in an unsystematic fashion, over an unspecified time, with unquantified results (Krasne et al., 2013).

Perceptual training, aimed at developing *perceptual* skills, has been applied to domains of human activity ranging from cricket play to language learning to airplane navigation (Tallal et al., 1996; Hopwood et al., 2011; Kellman and Kaiser, 2016). In the case of visual-based medical specialties, such as pathology, dermatology, and radiology, perceptual learning may be used to teach trainees how to visually recognize abnormalities, rather than how to interpret medical imaging based on a formal set of explicit rules (Chen et al., 2017). Yet, although this learning is a ubiquitous process in the adult brain, its implementation can require 10s of 1000s of trials of practice—without the certainty of learning success. Mere exposure to visual stimuli can produce perceptual learning, but it is often insufficient to yield robust results in the absence of additional factors, such as attention and reinforcement (Seitz, 2017).

Perceptual learning can be aided by training with attention. Covert attention, our ability to selectively process information at a given location without directing our gaze to that location, improves performance in many visual tasks mediated by basic visual dimensions, e.g., contrast sensitivity, spatial resolution and orientation (for reviews see Carrasco, 2011; Anton-Erxleben and Carrasco, 2013; Carrasco and Barbot, 2014). Research from the Carrasco lab has shown that covert attention can enable learning, in situations in which learning does not occur with training for the same time without attention (Szpiro and Carrasco, 2015), and facilitates transfer of learning to untrained locations (Donovan et al., 2015; Donovan and Carrasco, 2018). Possibly accounted for by plasticity in intermediate perceptual and higher decision-making brain regions, perceptual learning likely involves changes across a distributed collection of cortical areas (for review, Maniglia and Seitz, 2018). Although much of the research thus far has focused on low level perceptual discriminations, there is considerable evidence that perceptual learning is equally applicable to high-level, complex tasks (Kellman, 2013; Krasne et al., 2013).

Kellman and colleagues combined perceptual learning and adaptive learning methods (training that adapts in real-time to the trainees’ activity, and adjusts to their performance) to develop a web-based instruction program dubbed Perceptual and Adaptive Learning Modules (PALM), which does not rely on explicit instructions (Kellman et al., 2008; Rimoin et al., 2015). The PALM database includes multiple categories of diseases, and a typical module presents the learner with the task of discriminating or classifying a structure over a variety of instances, allowing the learner to discover the invariant properties of the given structure (Kellman et al., 2010). Mastery is gauged based on both accuracy and response time, given that experts are not only more accurate, but also able to complete tasks in a shorter time (and with less cognitive effort) than novices (Gunderman et al., 2001).

PALM has been successfully applied in medical perceptual training, including teaching histopathologic and dermatologic diagnosis to medical students, and echocardiography interpretation to anesthesiology residents. Learners demonstrated improvements in accuracy and fluency (sequential accurate responses made with short response times) even 6 months after training. Their training gains surpassed those typically obtained in traditional teaching (Krasne et al., 2013; Rimoin et al., 2015; Romito et al., 2016).

Sowden et al. (2000) conducted one of the first perceptual learning studies in radiologic imaging. They found that novice film readers improved their discriminations of clusters of microcalcifications in mammograms, and reduced their decision speeds, after following a perceptual learning regime where they viewed 60 images, three times each day, for 4 days. Negative feedback was provided in the form of a computer beep when the wrong cluster location was selected (Sowden et al., 2000). Remarkably, this work showed that, whereas radiologists in training may have already seen 1000s of images, even small amounts of practice in a relatively short interval, can produce significant improvements in sensitivity (Sowden et al., 2000; Krasne et al., 2013).

**FIGURE 8:**
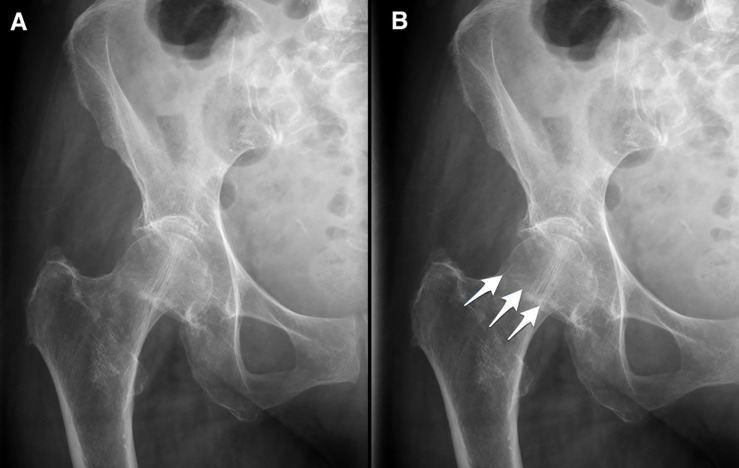
Reprinted from Chen et al. (2017) with permission. **(A)** Example of an image shown during perceptual training of hip fracture identification. **(B)** Arrows represent the feedback provided in case of a wrong answer. Top novices achieved expert level accuracy in hip fracture detection in under 1 h of perceptual training (Chen et al., 2017).

Chen et al. (2017) examined the efficacy of perceptual learning on the performance of novices with no prior knowledge of plain film interpretation, on the detection of hip fractures. They found that top performing novices achieved comparable accuracy to that of board-certified radiologists after 1280 images and 52 min of training. Whereas it is not known to what degree these findings might extrapolate to different pathology or imaging modalities, the data speaks to the potential of perceptual learning in radiology training (Chen et al., 2017) (Figure 8).

Books and digital resources abound to generate differential diagnoses for problematic findings (with pictures of representative abnormalities), but none of these resources address the first step of interpretation: perception. The fact that observational errors constitute the bulk of interpretive error in radiology (and that error rates have not changed in over half a century) (Waite et al., 2017b), highlights the need for (a) new educational methods to teach radiologists in training, and (b) the reassessment of present didactic and question-and-answer instruction techniques.

## A New Research Paradigm

As outlined in this review, there have been important strides in understanding the nature of a radiologists’ perceptual expertise. In short, experts possess more refined and complex search strategies, organize information more efficiently (Lesgold et al., 1988), are faster and in general make less perceptual errors than novices, partially because they are better able to discern lesions from the background. Yet, there is no concrete evidence that radiologists have superior perceptual skills outside of imaging, and as such there is no accepted or principled basis to choose radiology trainees that will be most likely to attain mastery. In addition, research has demonstrated that perceptual expertise peaks earlier than factual knowledge and begins very early in training (Ravesloot et al., 2017). Unfortunately, perceptual expertise also appears to plateau at a high level of error, accounting for the persistent high levels of error noted since Garland’s pioneering work in the 1940s (Garland, 1949).

Even with our current understanding of the nature of radiologic expertise, and how it develops during residency, significant gaps in knowledge remain. Importantly, these include translation of the knowledge base into concrete methods for radiologists to decrease interpretive error. Peak expertise is currently achieved by radiologists only after years of trial-and-error, resulting in the learning of hidden principles that have not yet been articulated, and as such are not explicitly recognized or consistently applied. Until we understand the precise perceptual criteria that radiologists apply to discriminate abnormalities in medical images, the field as a whole will not achieve peak performance.

The ability of a radiologist to see abnormalities largely depends on their skill to recognize subtle shapes and textures embedded in a noisy background. Radiologic expertise may therefore constitute the solution to a complex texture discrimination problem. If so, expert radiologists may learn to rapidly detect abnormalities in their peripheral vision, and then use this information to target central vision for deeper analysis. This idea differs from previous suggestions about the perception of textures in visual performance in radiology (Balas et al., 2009; Whitney and Yamanashi Leib, 2018) in that here we refer to texture in the most general possible sense. Although classical image statistics, such as contrast, entropy, and the correlation between central and nearby pixel intensities, are thought to guide ocular fixation targeting, these statistics are not necessarily task relevant and therefore do not provide a complete picture of the relationship between informativeness and ocular targeting (McCamy et al., 2014).

We hypothesize that it is the correlated combination of the cardinal dimensions—the basis of visual texture (Bergen and Adelson, 1988; Bergen, 1991)—that expert radiologists may use to detect abnormalities. That is, just as both woody and granite textures can share the same cardinal image statistics, they nevertheless appear dissimilar due to their different textures (they are visual metamers with respect to their first-order statistics, but not in their second-order statistics). Prior work on texture statistics in radiological diagnosis has focused on individual texture features (i.e., “co-occurrence”), or specific (non-general) types of potentially relevant textures to radiology, while ignoring or actively removing information from other aspects of texture (i.e., orientation) (Hu et al., 2016). Previous studies also suggest that adding specific textures to objects in films can increase their visibility (Vittitoe et al., 1997). However, these previous studies necessarily chose what they felt were relevant textures, and their choices were to some extent arbitrary (as there is no established principle to know which textures are critical). Here, we propose that the field of radiology should determine the relevant textures from the entire space of second-order statistics that the human visual system can perceive. This approach entails the employment of a general model of texture statistics (Heeger and Bergen, 1995; Portilla and Simoncelli, 2000; Freeman and Simoncelli, 2011) to ascertain empirically, from the entire space of possible textures, which features are diagnostically informative. Such a strategy is likely to result in a principled and non-arbitrary set of informative textures that represent the difference in perceptual abilities between experts and novices (Armato et al., 2011). Once known, specific perceptual learning heuristics could be designed to train for enhanced detection of those particular textures.

The proposed model would fit all known results to the best of our knowledge, and is supported by studies showing that experienced radiologists tend to have longer saccadic lengths between fixations, suggesting that they are better able to see targets further out in their peripheral vision (and use them to target subsequent fixations) than novices (Alzubaidi et al., 2010). Extensive training may enable radiologists to perceptually learn to detect abnormal textures at increased retinal eccentricities, earlier in their instruction and more consistently. It follows that analysis of fixation consistency across radiologists (a measure of image ‘informativeness’) may account for both bottom–up and top–down influences on image exploration (McCamy et al., 2014).

## Conclusion

Despite recent improvements in computer aided detection (CAD) and machine learning algorithms, radiologic interpretation is likely to remain a human task for the foreseeable future. Although many radiologists are concerned about artificial intelligence (AI) displacing them, most scholars are now of the opinion that AI will *augment* rather than replace radiologists (Liew, 2018). In a future where radiologists are mandatory as component human authorities (Liew, 2018), educational and practical interventions to improve human perceptual and decision-making skills continue to be needed to improve accuracy and reduce medical error (Waite et al., 2017b; Ekpo et al., 2018).

Radiology must move past the declarative knowledge paradigm to advance its training models and decrease its long-standing high error rate. In short, simply informing radiologists about potential errors does not improve their perception.

We propose that one way to improve radiologic instruction is, first, to determine precisely what makes abnormalities different from normal tissue—as in which textures are most informative—and then specifically train physicians to detect these textures with their peripheral vision, to enhance their ability to find abnormalities in medical images. This knowledge could afford focused perceptual learning, thereby supplementing conceptual knowledge and providing the exposure to abnormalities required for sensitivity improvements to occur during residency, rather than waiting (and hoping) for them to develop during routine radiologic practice. Our review of the literature moreover indicates the need for a deeper understanding of expertise-related individual differences in oculomotor-behavior, especially with regard to the informativeness of image regions. By precisely characterizing the contributions from each of these skills to the radiologist’s toolkit (and any potential overlap), we may be able to optimize heuristics for training on each of them.

As a field dominated by a primarily *perceptual* task, radiology needs a more refined understanding of perceptual expertise to improve accuracy, reduce error, and improve patient care.

## Author Contributions

SW conceived and wrote the manuscript. AG wrote an initial section of the manuscript and contributed to the figures and permissions. RA wrote a section of the manuscript. SM contributed to the editing of the manuscript. SM, MC, DH, and SM-C contributed to the conception of the work. SM-C was responsible for extensive editing. All authors contributed to the revising, reading, and approving the final version of the manuscript for submission.

## Conflict of Interest Statement

The authors declare that the research was conducted in the absence of any commercial or financial relationships that could be construed as a potential conflict of interest.
