# Corrigendum: Analysis of Perceptual Expertise in Radiology – Current Knowledge and a New Perspective

**DOI:** 10.3389/fnhum.2019.00272

**Published:** 2019-08-13

**Authors:** Stephen Waite, Arkadij Grigorian, Robert G. Alexander, Stephen L. Macknik, Marisa Carrasco, David J. Heeger, Susana Martinez-Conde

**Affiliations:** ^1^Department of Radiology, SUNY Downstate Medical Center, Brooklyn, NY, United States; ^2^Department of Ophthalmology, SUNY Downstate Medical Center, Brooklyn, NY, United States; ^3^Department of Neurology, SUNY Downstate Medical Center, Brooklyn, NY, United States; ^4^Department of Physiology/Pharmacology, SUNY Downstate Medical Center, Brooklyn, NY, United States; ^5^Department of Psychology and Center for Neural Science, New York University, New York, NY, United States

**Keywords:** visual perception, expertise, radiology, visual search, perceptual learning, attention, holistic processing, gist

In the original article, there was a mistake in the caption for **Figure 5**. The caption incorrectly states that the figure was from Reed et al. (2011) however the figure was reprinted from Litchfield and Donovan (2016) with permission. The correct legend appears below.

The authors apologize for this error and state that this does not change the scientific conclusions of the article in any way. The original article has been updated.

**Figure 5**. Reprinted from Litchfield and Donovan (2016) with permission. Two different experience groups—expert radiologists and psychology students—searched for lung nodules from CXR images using the flash-preview moving window (FPMW) paradigm. Participants looked at the target word for 15 s (“lung nodule”). They then saw a fixation cross for 200 ms, then either a mask preview (random array of colored pixels) or a CXR (a ‘scene’ preview) for 250 ms. Next, participants saw a mask for 50 ms and then a second fixation cross for 400 ms. Following a second presentation of the fixation cross, they conducted a windowed search, with a 2.5-degree radius window restricting the field of view (Litchfield and Donovan, 2016).
